# Circulating Tumor Cells in Melanoma Patients

**DOI:** 10.1371/journal.pone.0041052

**Published:** 2012-07-19

**Authors:** Gary A. Clawson, Eric Kimchi, Susan D. Patrick, Ping Xin, Ramdane Harouaka, Siyang Zheng, Arthur Berg, Todd Schell, Kevin F. Staveley-O’Carroll, Rogerio I. Neves, Paul J. Mosca, Diane Thiboutot

**Affiliations:** 1 Gittlen Cancer Research Foundation and Department of Pathology, Hershey Medical Center, Pennsylvania State University, Hershey, Pennsylvania, United States of America; 2 Materials Research Institute, The Pennsylvania State University at University Park, University Park, Pennsylvania, United States of America; 3 Department of Surgery, Hershey Medical Center, Pennsylvania State University, Hershey, Pennsylvania, United States of America; 4 Department of Bioengineering, The Pennsylvania State University at University Park, University Park, Pennsylvania, United States of America; 5 Division of Biostatistics, Department of Public Health Sciences, Hershey Medical Center, Pennsylvania State University, Hershey, Pennsylvania, United States of America; 6 Department of Microbiology and Immunology, Hershey Medical Center, Pennsylvania State University, Hershey, Pennsylvania, United States of America; 7 Division of Plastic Surgery, Department of Surgery, Hershey Medical Center, Pennsylvania State University, Hershey, Pennsylvania, United States of America; 8 Department of Surgery, Duke University, Durham, North Carolina, United States of America; 9 Department of Dermatology, Hershey Medical Center, Pennsylvania State University, Hershey, Pennsylvania, United States of America; Ospedale Pediatrico Bambino Gesù, Italy

## Abstract

Circulating tumor cells (CTCs) are of recognized importance for diagnosis and prognosis of cancer patients. With melanoma, most studies do not show any clear relationship between CTC levels and stage of disease. Here, CTCs were enriched (∼400X) from blood of melanoma patients using a simple centrifugation device (OncoQuick), and 4 melanocyte target RNAs (TYR, MLANA, MITF, and MIF) were quantified using QPCR. Approximately one-third of melanoma patients had elevated MIF and MLANA transcripts (p<0.0001 and p<0.001, respectively) compared with healthy controls. In contrast, healthy controls had uniformly higher levels of TYR and MITF than melanoma patients (p<0.0001). There was a marked shift of leukocytes into the CTC-enriched fractions (a 430% increase in RNA recovery, p<0.001), and no relationship between CTC levels and stage of disease was found. CTCs were captured on microfabricated filters and cultured. Captured melanoma CTCs were large cells, and consisted of 2 subpopulations, based on immunoreactivity. One subpopulation (∼50%) stained for both pan-cytokeratin (KRT) markers and the common leukocyte marker CD-45, whereas the second subpopulation stained for only KRT. Since similar cells are described in many cancers, we also examined blood from colorectal and pancreatic cancer patients. We observed analogous results, with most captured CTCs staining for both CD-45/KRT markers (and for the monocyte differentiation marker CD-14). Our results suggest that immature melanocyte-related cells (expressing TYR and MITF RNA) may circulate in healthy controls, although they are not readily detectable without considerable enrichment. Further, as early-stage melanomas develop, immature melanocyte migration into the blood is somehow curtailed, whereas a significant proportion of patients develop elevated CTC levels (based on MIF and MLANA RNAs). The nature of the captured CTCs is consistent with literature describing leukocyte/macrophage-tumor cell fusion hybrids, and their role in metastatic progression.

## Introduction

The ability to measure circulating tumor cells (CTCs) represents a potentially powerful method for monitoring patients with known malignancies with minimal morbidity. Many studies have demonstrated the diagnostic and prognostic value of CTCs in cancer patients, with much of the work focused on CTCs as biomarkers in breast and prostate cancers [1], [2]. The prognostic information inherent in CTC levels is only just beginning to be understood and the methods by which CTCs should be monitored are still evolving. Despite these uncertainties, emerging data indicate that monitoring CTCs will play an increasingly significant role in the diagnosis and treatment of cancer patients. While work has often demonstrated a relationship between CTC levels and cancer stage, it is also clear that a significant proportion of even early-stage cancer patients have detectable CTC levels. Since metastasis is a relatively inefficient process, we believe that CTCs can effectively be used to screen for early stage cancers. Toward this end, we are developing a chip-based sensor platform for CTC measurement [3], which will allow multiplexed simultaneous detection of target marker RNAs for a variety of cancers.

Melanoma is one of the most common cancers and it is increasing at a faster rate than any other malignancy. Approximately 76,250 new melanomas will be diagnosed and 9,180 people are expected to die from melanoma in the United States in 2012. With a large number of new patients and survivors, routine surveillance of this population presents a significant logistical and financial challenge. Patients who are at highest risk for recurrence require not only routine skin examinations, but also costly and potentially harmful radiologic imaging (PET/CT scans and brain MRI). These methods are effective in finding recurrences and new melanomas once they are visible on imaging or on dermatologic exam, but monitoring CTCs in these patients may provide important prognostic and diagnostic information at an earlier time than traditional methods. In particular, CTCs in melanoma have engendered considerable interest [4], [5], [6], [7], [8]. The currently approved measurements for CTCs utilize CellSearch (Veridex), but most experimental studies have been conducted using QPCR for various melanoma marker RNAs. Tyrosinase (TYR) has been the most widely used marker RNA [5], [9], [10], [11], [12], [13], along with Melan-A/MART-1 (MLANA) [6], [9], [12], [13], [14], [15], and a comparative survey identified MLANA and TYR as “optimal” markers [11]. Another less frequently studied marker is microphthalmia-associated transcription factor (MITF) [10], [15], which is a transcription factor controlling expression of genes involved in melanogenesis. We also focused on one additional potential marker, the cytokine known as macrophage migration inhibitory factor (MIF). MIF has been identified as a serum marker for melanoma [16], and MIF is also elevated in colorectal cancer [17], [18] and a variety of other types of cancer [19]. MIF plays a role inflammation in addition to tumorigenesis [20], and its growth-promoting properties involve the AKT pathway [19]. Studies in general have not demonstrated a clear relationship between melanoma stage and CTC levels (see for example [5]), and it is clear that a portion of patients with very early stage cutaneous melanomas have relatively high levels of CTCs [5], [14], and such patients are at risk for subsequent development of metastatic lesions [14].

Here we have performed CTC measurements on a series of melanoma patients (most were early stage patients). We employed a simple porous membrane gradient centrifugation device, OncoQuick [21], [22], [23], to isolate a fraction from whole blood which is enriched ∼400–500X for CTCs (OncoQuick enrichment is based on CTCs having a lighter buoyant density than peripheral blood mononuclear cells, PBMCs), and then isolated RNA and measured melanoma marker RNAs using QPCR. Following quantification of CTCs, we then captured CTCs from melanoma patients using OncoQuick in conjunction with a filter-based device, and grew them in culture for preliminary marker characterizations. Given our initial staining results with melanoma samples, we then also captured and characterized CTCs from colorectal and pancreatic cancer patients.

## Results

**Figure 1:**
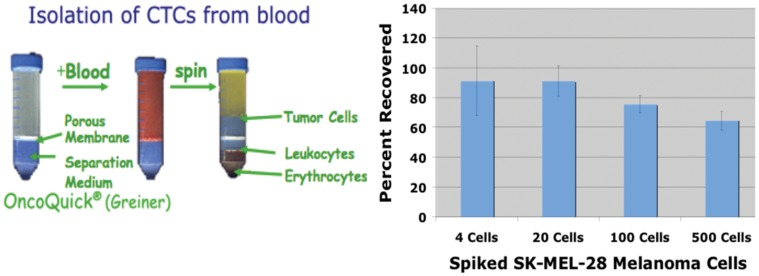
OncoQuick Enrichment and Recovery of Melanoma CTCs. Left Panel shows a schematic of OncoQuick enrichment. Right Panel shows recovery of SK-MEL-28 human melanoma cells from blood. Known numbers of SK-MEL-28 cells were added to 7.5 ml of blood, and processed using OncoQuick spin columns as described. Recovery of cells was assessed using QPCR amplification of KRT8 and 18 RNAs as described.

In preliminary spiking experiments using the OncoQuick columns, we observed >90% recovery of spiked SK-MEL-28 cells with 4 or 20 cells added (Figure 1); recovery decreased slightly with 100 to 500 cells added; the basis for this decrease (which sometimes reaches borderline statistical significance) is not clear, but may involve aggregation or clumping). We have observed the same recovery pattern with breast, colorectal, pancreatic, and prostate cancer cells (data not shown).

We then obtained blood samples from melanoma patients with various stages of disease, as well as blood samples from healthy control subjects. Blood samples were fractionated using the OncoQuick columns and RNA was purified from the enriched CTC fractions.

We observed a marked increase in RNA in the enriched CTC fractions from melanoma patients (Table 1), which amounted to a 430% increase in RNA. This presumably represents RNA from PBMCs, which have shifted into the lighter density fraction in which the CTCs are found (see Discussion).

**Table 1:** RNA recoveries in the “Enriched CTC” fractions in melanoma patients vs. healthy controls.

	Melanoma Patients	Healthy Controls
Mean (pg/ml)	2512	471
Std. Dev.	2560	528

There is a statistically significant difference between RNA yield levels of melanoma patients and the healthy controls (p = 0.005) based on the nonparametric Wilcoxon rank-sum test. There was no statistically significant correlation between melanoma stage and RNA yield. This was true for scale 1 (stage 1,2,3, or 4; p = 0.739) and scale 2 (where the scale separates 0, IA, IB, IIA, IIB, IIC, etc.; p = 0.948).

**Figure 2:**
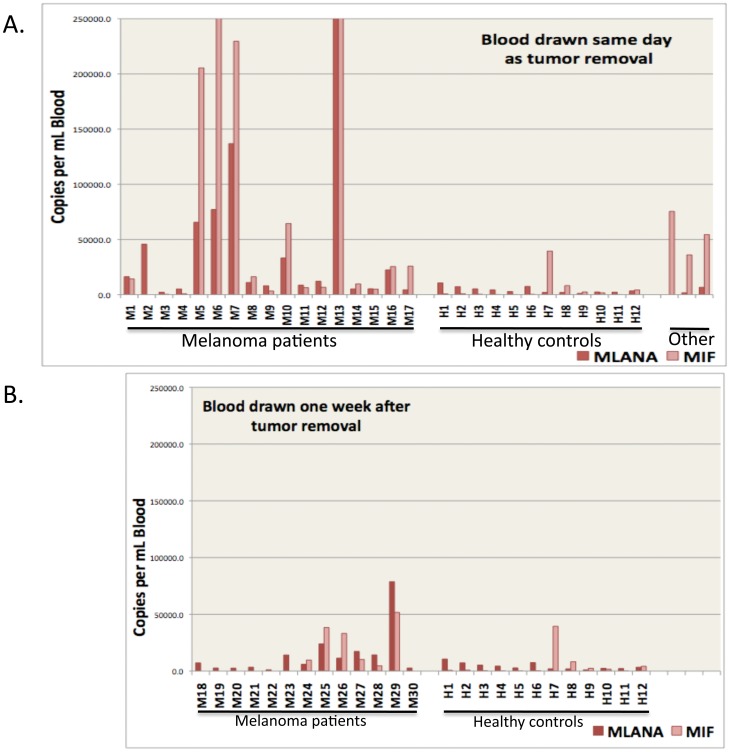
Marker RNA levels for MLANA and MIF in melanoma patients vs. healthy controls. Blood was fractionated as described using OncoQuick columns, RNA was purified from the enriched CTC fractions, and MLANA and MIF RNA levels were quantified by QPCR. Panel A shows results with blood drawn on the same day as excision of melanomas, or the corresponding healthy controls. Panel B shows results with blood drawn one week after excision. Melanoma patients and healthy controls are as noted on the X-axis, and Staging information for melanoma patients is presented in Table 2. The Y-axis depicts RNA copy numbers per ml of blood. Shown to the far right of Panel A are calculated transcript numbers for 2 patients with squamous cell carcinoma of skin and for a patient with neurofibromatosis (left to right, respectively).

**Figure 3:**
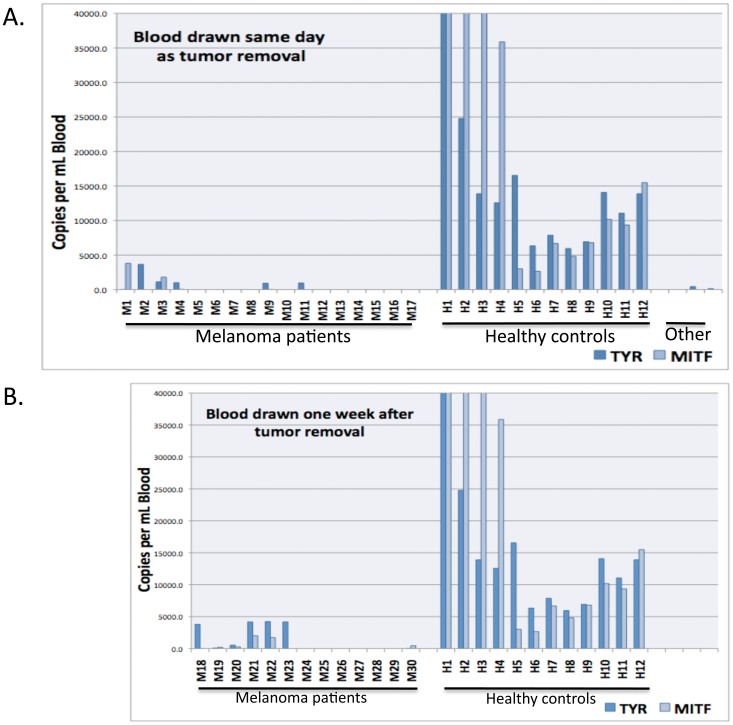
Marker RNA levels for TYR and MITF in melanoma patients vs. healthy controls. Blood was fractionated as described using OncoQuick columns, RNA was purified from the enriched CTC fractions, and TYR and MITF RNA levels were quantified by QPCR. Panel A shows results with blood drawn on the same day as excision of melanomas, or the corresponding healthy controls. Panel B shows results with blood drawn one week after excision. Staging information for melanoma patients is given in Table 2. The Y-axis depicts RNA copy numbers per ml of blood. Shown to the far right of Panel A are calculated transcript numbers for 2 patients with squamous cell carcinoma of skin and for a patient with neurofibromatosis (left to right, respectively).

Target RNA transcript levels (MLANA, MIF, TYR, and MITF) were then quantified by QPCR. With the MLANA and MIF targets, elevated levels were observed in a number of melanoma patients compared with healthy controls (Figure 2). Substantial increases in MLANA transcripts were observed in ∼ one-third of melanoma patients, accompanied by increases in MIF. This was true for blood samples drawn at the time of surgery, as well as samples drawn one week following surgery. After applying a logarithmic transformation, a two sample t-test was used to identify statistical differences between the healthy volunteers and melanoma patients. Both MLANA and MIF were significantly higher in melanoma patients (p<0.013 and p<0.0001, respectively). In contrast, transcript levels were significantly higher for TYR and MITF (each p<0.0001) in blood from healthy control subjects vs. melanoma patients (Figure 3), again both in blood samples drawn at the time of surgery as well as those drawn one week following surgery.

Using a test of Pearson’s product moment correlation coefficient no significant correlation was found between any of the four biomarkers and stage of melanoma, and there was no correlation between RNA yield and stage (Table 1).

Highly significant correlations generally exist between the various markers (p<0.0001), with a slightly lesser significance between MLANA and MITF (p = 0.005), with the exception being between MLANA and TYR markers, where no statistical significance was found (p = 0.11).

**Figure 4:**
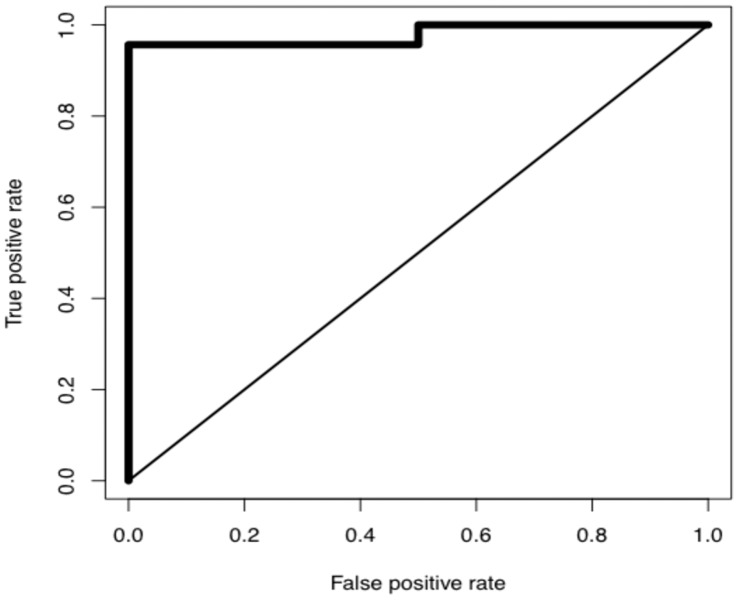
Receiver Operating Characteristic Analysis for MIF. ROC analysis was performed using the R Package software version 1.0-4 as described [53].

**Figure 5:**
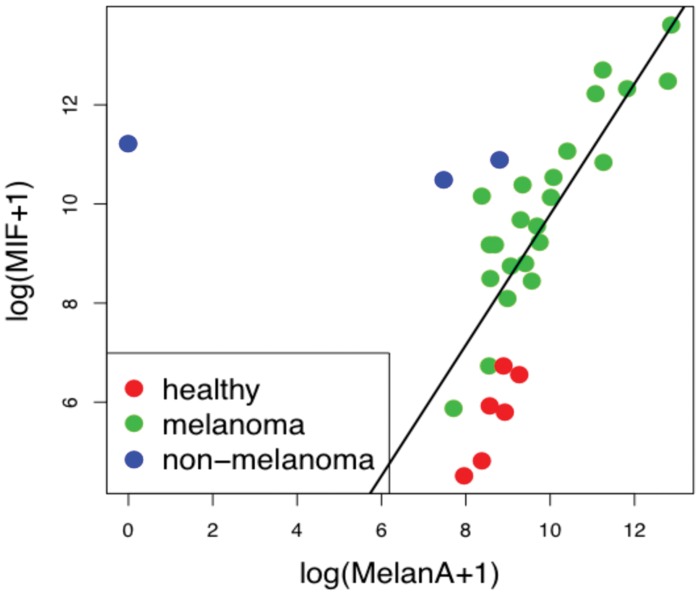
Correlation between MIF and MLANA Transcript Levels. MLANA and MIF transcript levels were quantified using QPCR, and the correlation between them was calculated using the R software program version 2.13.1.

Receiver operating characteristic (ROC) curves provide a graphical tool to assess the diagnostic utility of markers, and are often applied to melanoma markers, recent examples including autoantibodies [24], circulating cell-free DNA amplicons [25], and MIA [26]. A Receiver Operating Characteristic analysis for MIF showed an Area Under the Curve of 0.987 (Figure 4), and a highly significant correlation was found between MIF and MLANA (Figure 5; p<0.0001).

However, in spite of the high correlation between MIF and MLANA levels, there was also an interesting difference in the time course of expression. We compared 2 groups of melanoma patients for changes in absolute MLANA levels vs. MIF levels based on time following surgery. One group of patients (n = 17) had their blood drawn and analyzed on the day of melanoma removal surgery, whereas a 2^nd^ group of patients (n = 13) had their blood drawn and analyzed 1 week following the surgery. Using a two-sample t-test comparison of the log-transformed MIF transcript levels, no differences were noted in blood taken on the day of surgery vs. levels in patients one week following surgery (p = 0.99). However, a decline was observed in MLANA levels (with borderline significance, p = 0.05). These data seem to suggest that MLANA may be a relatively sensitive and specific marker for melanoma CTCs, and that MLANA levels decay relatively quickly upon removal of the lesion, whereas MIF is associated with a broader spectrum of processes (cancer, inflammation, etc.) and seems to decline on a more protracted time-scale (if at all).

We then cultured CTCs from melanoma patients, using OncoQuick enrichment in conjunction with a filter device which was designed to capture the larger CTCs based on size [27]. We were able to capture large CTCs from 2 of 4 early-stage melanoma patients on the filters, although they grew very slowly if at all; after 6 weeks in culture, there was no obvious increase in cells on the filters. The captured cells consisted of large cells (generally 20–30 µm).

**Figure 6:**
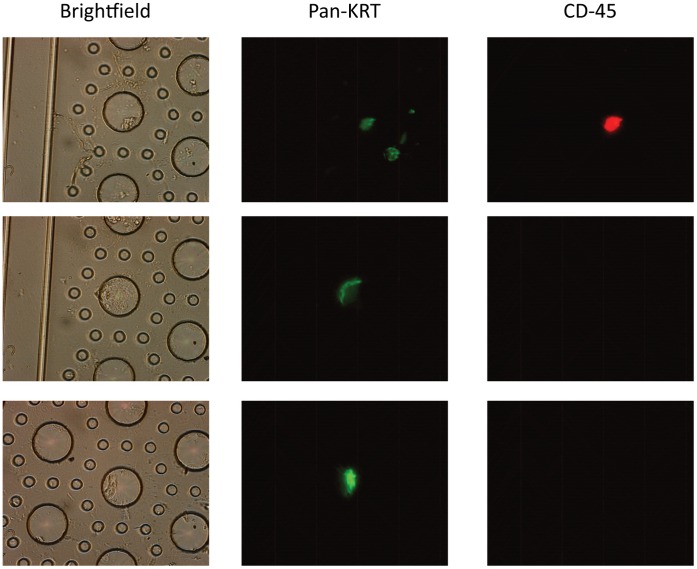
Representative Melanoma CTCs Captured on a “Splitable Gap” Filter Device. CTCs were enriched from blood from melanoma patients using OncoQuick and the enriched CTC fraction was then passed through the filter device. Filters containing captured cells were then placed in culture for various times up to ∼ 42 days, and cells were subsequently stained with DAPI, and stained for immunofluorescence using pan-KRT and CD-45 antibodies as described. Left panels show brightfield pictures of cells within wells of the filter; large wells are 50 µm in diameter, whereas the surrounding smaller pores are 7.5 µm in diameter. Center panels show immunofluorescent staining of cells with for pan-KRT marker, and right panels show staining for the common leukocyte antigen CD-45. All cells shown stained positively with DAPI.

Given the apparent aberrant expression of typical melanocytic differentiation markers (such as TYR, MLANA), we chose to stain potential melanoma CTCs for pan-KRT, which is generally expressed in melanomas and has prognostic significance (particularly KRT18; see Discussion). We observed 2 subpopulations based on immunofluorescent staining characteristics (Figure 6). One subpopulation (∼50%) of cells stained for both pan-KRTs and for CD-45, the standard common leukocyte marker for PBMCs, The other subpopulation stained only for pan-KRTs (Figure 6).

**Figure 7:**
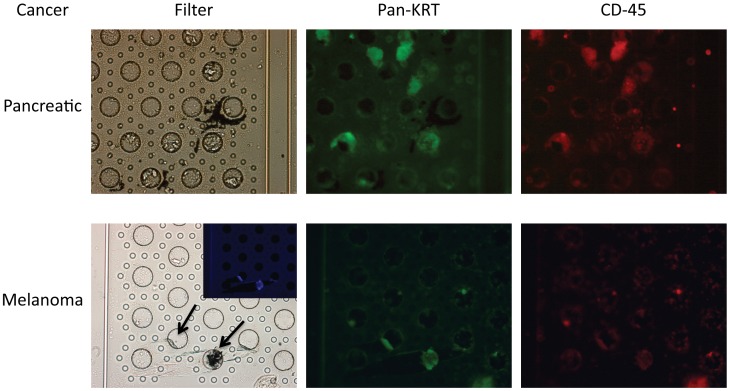
Melanoma and Pancreatic CTCs Captured on Filters. CTCs were enriched from blood samples from another melanoma patient and from a patient with pancreatic cancer using OncoQuick, CTCs from the enriched fraction were captured on filters, and cells were cultured as described. Upper row shows a brightfield shot of pancreatic CTCs growing on the filter, and immunofluorescent staining of CTCs for pan-KRT and CD-45 (as indicated). Lower row shows CTCs from another melanoma patient. Inset in the brightfield panel shows DAPI staining for 2 of the cells on the filter (the 3^rd^ cell also stained positively, not shown), which was difficult to photograph due to inherent fluorescence from the filters.

Cells similar to the captured CTCs have recently been described in histologically negative sentinel lymph nodes [28]. Itakura et al. described 2 subpopulations of melanoma-related cells, which seem analogous to the 2 subpopulations of cells we report here from blood. One subpopulation was composed of cells consistent with immature melanocytes, which expressed MLANA mRNA but not MLANA protein. The other subpopulation also contained MLANA mRNA, but also expressed leukocyte/macrophage markers. Given the considerable body of literature describing leukocyte/macrophage-tumor cell fusion, both in melanomas and in many other cancers (see Discussion), we also examined blood from colorectal (3) and pancreatic (1) cancer patients, and were able to capture CTCs from all of them. The CTCs from colorectal and pancreatic cancer patients did grow in culture, albeit slowly, in contrast to melanoma cells (which were maintained but did not appear to multiply). The proportion of cells dual-staining for Pan-KRT and CD-45 was higher for pancreatic CTCs; essentially all cells dual-stained for pan-KRT and CD-45 (Figure 7).

**Figure 8:**
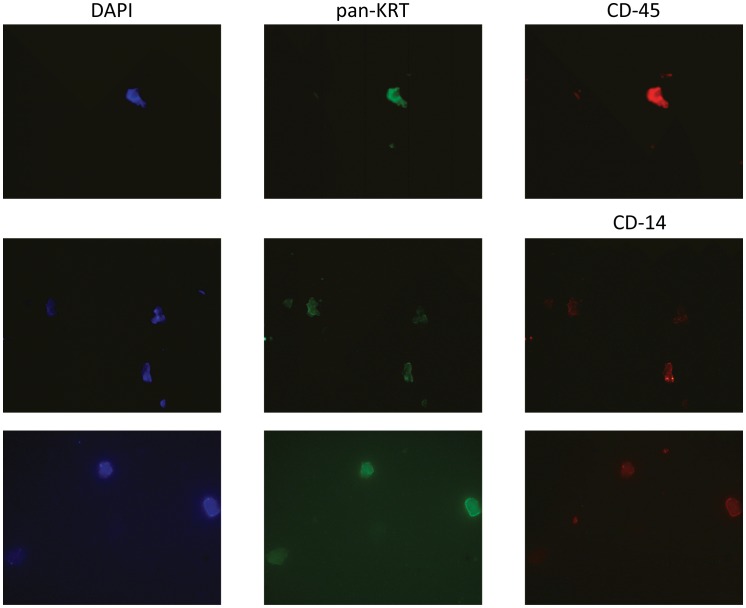
Colorectal CTCs Enriched Using OncoQuick and Cultured Directly. CTCs were enriched from blood from 2 patients with colorectal cancer using OncoQuick. The enriched CTC fractions were then plated in culture and grown as described. Top row shows DAPI staining and immunofluorescent staining for pan-KRT and CD-45 markers. Lower 2 rows show DAPI staining, and immunofluorescent staining for pan-KRT and the macrophage differentiation marker CD-14. All 3 cells in the middle row stained positively for both pan-KRT and CD-14, and 2 of the 3 cells shown in the lower row stained positively for pan-KRT and CD-14.

We also obtained enriched CTC fractions from 2 patients with colorectal cancer (using OncoQuick), and cultured them directly (i.e., not using the filter device). The cultured colorectal CTCs were again relatively large cells, similar in appearance to those isolated with the filter device. These rare colorectal CTCs also showed dual-staining for pan-KRT and CD45 (Figure 8). In addition, one preparation was also stained for the monocyte differentiation marker CD-14; the cultured colorectal CTCs stained positively for CD-14, as well as for pan-KRT (Figure 8). CellSearch analyses for these same 3 colorectal patients was negative for 2 of the patients, and reported 1 CTC for the third patient.

## Discussion

It is becoming clear that CTCs are found in a significant subset of patients with early-stage melanomas [8], and it has been shown that CTC levels have prognostic significance [12], [29]. Our results indicate that ∼1/3 of melanoma patients (nearly all of them early stage) have significantly elevated levels of MLANA and MIF marker RNAs compared with healthy volunteers, and similar results have been reported in a number of other studies [12], [14], [29], [30]. Elevated MLANA and TYR levels from samples taken in a post-operative period have been reported to identify ∼ two-thirds of early stage patients who went on to develop disseminated or locally recurrent disease [14], with the caveat that MLANA expression was significantly lower in the patients with disseminated disease. It therefore seems reasonable to begin to evaluate MLANA expression, and potentially MIF expression, on a large-scale prospective basis, in an attempt to identify early-stage patients who may warrant more aggressive therapy, as well as following their levels during and after therapies.

Our data also showed 3 striking findings. The first pertains to the TYR and MITF markers, which reflect components involved in the early stages of melanogenesis. These were highly significantly elevated (p<.0001) in normal healthy volunteers, compared to patients with melanomas. Others have also reported finding TYR (and also MLANA) in blood from healthy controls [11], but most reports are negative. We point out that here we initially used OncoQuick devices, which enriched the relative CTC levels by a factor of ∼400–500. It seems reasonable to propose that this enrichment, which is the equivalent of 9–10 rounds of reasonably efficient PCR amplification, may have shifted TYR (and MITF) from undetectable/borderline levels to detectable levels. However, the striking finding was that TYR and MITF transcripts were not detectable in patients with melanomas. As a potential explanation, we hypothesize that melanocyte precursor cells – cells which express TYR and MITF, but not MLANA (or MIF) - may circulate in healthy individuals. There is some evidence showing that melanocyte precursor cells which express MITF and TYR are present in human cord blood [31], and in a potentially related vein induction of pluripotent stem cells and subsequent melanocytic differentiation in culture produces cells expressing MITF and TYR [32]. With the development of melanomas, it is possible that factors (perhaps cytokines including MIF, etc.) are systemically released by tumors (or the tumor microenvironment) which inhibit entry of these normal melanocyte precursors into the circulation, and that melanoma cells expressing MLANA and MIF (but not TYR or MITF) begin to enter the circulation. While MIF transcripts may serve as a good marker for early stage melanoma, the sources of MIF are not clear; MIF is also elevated in the SCC/NF patient samples. Why some melanoma cells appear to express MLANA, but not TYR or MITF, is also not clear, but this hypothesis leads to the prediction that TYR and MITF levels will increase to control levels with time after removal of the melanomas. In this regard, TYR and MITF levels did appear to increase in the blood samples drawn one week following surgery vs. samples drawn on the day of surgery, although the amount of skewness in the data limited the power to detect a statistically significant difference.

Another surprising finding was the major increase in RNA recovered from the CTC-enriched fractions in melanoma patients vs. healthy volunteers, which amounted to an ∼430% increase in RNA (p = 0.005). This clearly indicates a significant shift of PBMCs into the lighter density range characteristic of CTCs, although the underlying causes are unclear.

We then went on to capture and culture CTCs from melanoma patients (Figures 6 and 7). The putative CTCs consisted of large cells, about half of which stained for both pancytokeratins (pan-KRT) and for CD-45. These captured/cultured melanoma CTCs appear to be analogous to the melanoma-related cells recently been reported in “histologically negative” sentinel lymph nodes [28]. These authors described MLANA-RNA-expressing cells which appeared consistent with melanoma cells, as well as putative macrophage-melanoma hybrids. Such “hybrid” melanoma cells have also been reported in a number of other studies [33], [34], [35], [36], [37], [38], although this is the first report describing them in peripheral blood.

For detection of melanoma CTCs, we chose to use KRT as a marker, given the aberrant expression of typical markers of melanocytic differentiation. It is now widely recognized that melanoma cells generally express KRTs (mRNA and protein). In our hands, all melanoma cell lines we generally use in the lab strongly express KRT18 (data not shown), and similar reports for KRT18 expression in cell lines are also in the literature [39]. While IHC staining for KRT is often infrequent in routinely processed melanoma specimens, expression is far higher in fresh specimens [40], which appears to reflect selective destruction of the alpha-helical regions within KRT molecules during FFPE processing, complicating interpretation of the literature with respect to KRT expression in melanoma. KRT expression has long been related to migration, invasion, and progression [41], [42]; some recent work with melanoma cell lines has supported such a role [43], and KRT18 mRNA detection is associated with a poor prognosis in melanoma cases [39].

In fact, there is a body of literature indicating that leukocyte-tumor cell fusion may play an important role in tumorigenesis in many types of cancer [34], [44], [45], [46], [47], [48]. Based on this literature, and given our melanoma CTC results, we went on to capture and culture CTCs from blood from pancreatic (Figure 7) and colorectal (Figure 8) cancer patients, using either OncoQuick enrichment in conjunction with a microfilter device, or OncoQuick enrichment with direct plating/culture. We observed analogous large CTCs, which also showed dual-staining for pan-KRT/CD-45, with both colorectal and pancreatic cancer patients. In fact, the proportion of cells showing dual-staining appeared to be considerably higher in colorectal and pancreatic cancer CTCs (for pancreatic cancer CTCs perhaps 100%, although the numbers of captured cells were small). In a further experiment, we also enriched/cultured colorectal CTCs from another colorectal cancer patient, and demonstrated that the all of the cells also stained positively for the macrophage differentiation marker CD-14. It is not clear whether the dual-staining population may have selectively increased during growth in culture, although this did not appear to be the case in melanoma samples, since little or no growth occurred even with prolonged culturing.

These data pose problems and controversies for evaluation of CTCs. First, dual-staining pan-KRT/CD-45 cells are eliminated from consideration by the CellSearch system, which automatically rejects CD-45-positive cells. In fact, CellSearch analyses of the colorectal cancer patients showed a single CTC in one patient, and no CTCs in the other 2 patients. CD-45 positivity in the dual-staining KRT/CD-45 cells is also unfortunately problematic for “negative selection” approaches [49], which utilize CD-45 expression to eliminate “normal” cells.

## Materials and Methods

All human studies were conducted in accordance with principles outlined in the Declaration of Helsinki and were approved by the Institutional Review Board of Pennsylvania State University (Protocol #25070EP), with a matching protocol approved at Lehigh Valley Hospital. Informed consent was obtained in writing from all participants. De-identified samples (generally 7.5 ml) were obtained from melanoma patients and healthy volunteers (34 females, 11 males). The demographics for our patient population are 97% Caucasian, 2% Hispanic, and 1% African American, and most patients are between 30–60 years of age for superficial spreading melanomas. Blood was drawn from patients either on the day of surgery (just before surgery), or at follow-up visit 1 week after surgery (Patient numbers and their corresponding AJCC staging are given in Table 2). The staging classifications for samples where blood was drawn on the same day as melanoma removal encompassed 7 T1 samples, 2 T2 samples, 4 T3 samples, and 3 T4 samples (with 1 Tx sample). Staging classifications for samples where blood was drawn 1 week after surgery encompassed 11 T1 samples, 1 T2 sample, and 1 T4 sample.

**Table 2:** AJCC Stage of Melanoma Patients.

Study Participant	Stage
M1	Tx, N2a, M0
M2	T1a, MX, NX
M3	T1a, MX, NX
M4	T1a, MX, NX
M5	T1, MX, NO, R1
M6	T1a, MX, NX
M7	T1a
M8	T1b, R1
M9	T2a, MX, NX
M10	T2b, N1a, M0
M11	T3a, N3, M0
M12	T3a, N3, M0
M13	T3b, MX, NX, R1
M14	T3b, N2a, M0
M15	T4b, N0, M0
M16	T4b, N2a, M0
M17	T4b, NX, M1
M18	T1a, M0, N0
M19	T1a, M0, N0
M20	T1a, M0, N0
M21	T1a, M0, N0
M22	T1a, M0, N0
M23	T1a, M0, N0
M24	T1a, M0, N0
M25	T1a, M0, N0, R0
M26	T1, M0, N0, R0
M27	T1
M28	T1a, M0, N0, R0
M29	T2a, MX, NX, N0, RX
M30	T4b, M0, N0

Blood samples were processed within 24 h after collection using OncoQuick columns as per manufacturer’s recommendations, using 7.5 ml blood +7.5 ml wash buffer (PBS +0.5% BSA). OncoQuick enrichment is based on the fact that CTCs have a lighter buoyant density than PBMCs, so that they remain on top of the liquid (of defined density) used for the separation. Columns were centrifuged at 1,600×g for 20 min at 4 C (with slow acceleration). The liquid above the porous barrier was carefully removed and transferred to a 50 ml conical centrifuge tube, and the inner OncoQuick tube wall and porous barrier were washed 3X with 5 ml wash buffer, which was then withdrawn and transferred to the same conical centrifuge tube. The conical centrifuge tube was then centrifuged at 200×g for 10 min at 4 C. About 45 ml of the supernate was withdrawn and discarded, and 45 ml wash buffer was added, mixed, and the tube was re-centrifuged as before. The pellet, which we refer to as the “Enriched CTC” fraction, was resuspended in 600 microliters RLT lysis buffer for isolation of RNA. RNA yield was calculated using a Nanodrop instrument, and RNA Integrity Number (RIN) was assessed using a BioAnalyzer (Agilent). We used 1/10 of the sample for QPCR analyses and extrapolated results to calculate copies of RNA/ml of blood.

For spiking experiments (3 independent experiments), known numbers (4, 20, 100, or 500) of SK-MEL-28 cells (HTB-72, from ATCC) were added to 7.5 ml blood samples, and processed in the same manner. For these spiking experiments, rabbit blood (which was otherwise to be discarded) was generally used. It was collected under an approved IACUC protocol (#2005-147), which followed institutional guidelines. Control aliquots were harvested by centrifugation without OncoQuick fractionation. Recovery of cells was quantified based on recovery of cytokeratin 8 (KRT8) and 18 (KRT 18; see below). Recoveries of cells spiked into rabbit blood do not differ from those obtained using patient blood.

### QPCR

The 4 target genes we tested were: Tyrosinase (TYR, Gene ID#7299), macrophage migration inhibitory factor (MIF, Gene ID#4282), Melan A (MLANA, Gene ID#2315), and microphthalmia-associated transcription factor (MITF, Gene ID#4286).

Primers and TaqMan probe sequences used for QPCR were as follows:

MLANA forward primer was 5′-TTTCGCTTTTGTTGCCCAG, MLANAr was 5′-TAATCCCAGCTACTCAGGAGGCTA, and the TaqMan probe was 5′-FAM -CTGGAGTGCAATGGCGCGATCTT -BHQ-1-3′ (6-carboxyfluorescein  =  FAM, Black Hole Quencher 1 = BHQ1).

TYR forward primer was 5′-GGCTGTTTTGTACTGCCTGCT, TYRr was 5′-AGGAGACACAGGCTCTAGGGAA, and the TaqMan probe was 5′-HEX- AGTTTCCAGACCTCCGCTGGCCA-BHQ-1-3′ (CAL Fluor orange 560 = HEX).

MITF forward primer was 5′-TCAGCAACTCCTGTCCAGCC, MITFr was 5′-CCTCTCTTTGGCCAGTGCTC, and the TaqMan probe was 5′-Cy5- CCCAACATAAAAAGGGAGCTCACAGCG) BHQ-2-3′ (Quasar 670 = Cy5).

MIF forward primer was 5′-AAGCTGCTGTGCGGCCT, MIFr was 5′-TTGTTCCAGCCCACATTGG, and the TaqMan probe was 5′-Cy5- CGCATCAGCCCGGACAGGGT-BHQ-2-3′.

For the cytokeratins used in the spiking recovery experiments, Cytokeratin 8 (KRT8) forward primer was 5′-TCCTCACCAAGAAGCAGCTTC, KRT8r was 5′-TGGGTCACCCTGATGGACAT, and the TaqMan probe was 5′-HEX-CTCCTTCTAGGATCTCCGCCTGGTTCG-BHQ-1-3′.

KRT18 forward primer was 5′AGATCGAGGCTCTCAAGGAGG, KRT18r was 5′-CACGGTCAACCCAGAGCTG, and the TaqMan probe was 5′-FAM-CGAAGAGGAAGTAAAAGGCCTACAAGCCCA-BHQ-1-3′.

QPCR was performed using a Stratagene Mx3005P machine as previously described [50], [51]. Samples were run in duplicate, and data were analyzed using the Stratagene MXPro software for absolute quantification. Standard curves were run using in vitro transcribed RNAs, as were negative controls.

### Statistical Analyses

The statistical software program R version 2.13.1 (R Development Core Team, 2011) was used to perform the analyses of RNA copy numbers and create graphics. The R package ROCR (version 1.0-4; [52]) was used to perform the receiver operating characteristic (ROC) analyses. A nonparametric Wilcoxon rank-sum test was used to compare RNA yield levels from melanoma patients vs. healthy controls.

### Cell Culture and Immunofluorescence Microscopy

Blood samples were collected and processed using OncoQuick columns as described. The supernatant fluid was carefully removed, and the cells in ∼1 ml of plasma were processed through a “splitable 3D parylene C membrane filter” [27]. Basically this microfabricated device has an integrated 3D structure of two layers of parylene membrane. The top layer contains large 50 µm diameter wells, while the bottom layer has smaller 7.5 µm diameter pores positioned around the rim of the large wells. The top layer is separated from the bottom layer by a gap of 5.5 µm, which is precisely defined during the microfabrication process, and furnishes an effective geometry for filtration. This device was engineered so that PBMCs could efficiently pass through the large wells, as well as through the small pores and the gap between the top/bottom layers. The larger epithelial cells could only enter the large 50 µm wells but not exit through the gap between the layers. In initial experiments, the filters containing the captured melanoma cells were cultured in ATCC melanocyte medium. In later experiments, a 50%/50% mixture of DMEM and RPMI-1640 was used, supplemented with 10% fetal bovine serum, 3 mM L-glutamine, and penstrep antibiotic. With colon and pancreatic CTC samples, the cells with 1 ml of plasma were removed as before, and resuspended in RPMI-1640 medium and plated for 2 h or overnight, and the medium was then changed and supplemented with M-CSF to a final concentration of 50 ng/ml for the remainder of the culturing (up to ∼50 day).

For immunofluorescence staining, CTCs were plated in medium in an 8-well coated chamber slide (Lab-Tek II CC^2^) and incubated overnight (O/N) at 37°C. On the following day, media was removed from cells by inversion-wicking with paper towels, and cells were rinsed with 1X PBS. 10% Neutral Buffered Formalin (NBF) was added to just cover the cells (∼200 µL), and slides were incubated at room temperature (RT) for 20 minutes. The cells were washed 3 times (2 minutes each wash) with 1X PBS, and washes were removed by inversion-wicking. Cells were permeabilized with ice-cold sodium citrate buffer (0.1% sodium citrate +0.1% Triton X-100 in 10% PBS) for 30 min on ice. Cells were again washed 3 times with 1X PBS and dried by inversion-wicking. Cells were then blocked in 2.5% bovine serum albumin in PBS-T (1x PBS +0.2% Triton X-100) for 1 hour at RT.

Primary antibodies (1° Abs) were diluted to the desired concentrations in the same blocking solution. 200–300 µL of the 1°Ab solution were added to each chamber-well, and the slides were incubated for 1–3 hours at RT (or in some cases at 4°C overnight in a humidifying chamber). After incubation with the 1° Ab solution, cells were washed 3 times (3 min each) with PBS-T. After this, all steps were performed in the dark.

2° Ab solutions were diluted to the desired concentration in blocking solution. 200 µL was added to each chamber-well, and incubation was for 45–60 min at RT. After incubation with the 2° Ab solution, the cells were again washed 3X (3 min each) with PBS-T (1x PBS +0.2% Triton X-100). To counterstain nuclei, DAPI was diluted in 1X PBS (1∶30,000). ∼200 µL DAPI solution was added to each chamber-well, and the slide was incubated for 5 min at RT in the dark. An additional wash in PBS-T was performed for 10 min at RT, and this was removed by inversion-wicking. Chambers were removed and separated with a tool provided by the manufacturer. Coverslips were mounted with 3 drops of ProLong Gold Antifade mounting media at RT. Slides were examined using fluorescence microscopy. Slides were stored at 4°C in the dark.

Antibodies used were as follows:

PanCytokeratin (pan-KRT) rabbit polyclonal (from Santa Cruz, #SC-15367) was used with Alexafluor-labeled donkey anti-rabbit 488 antibody (from Invitrogen, #A21206). This pan-KRT antibody is broadly reactive with human KRT family members. For detection of the common leukocyte antigen CD-45 (officially known as PTPRC, Gene ID#5788), a rat monoclonal antibody (from Santa Cruz, #SC59071) was used with Alexafluor-labeled goat anti-rat 568 antibody (from Invitrogen, #A11077). For detection of the monocyte differentiation antigen CD-14 (Gene ID#929). a mouse monoclonal antibody (from BD Pharmingen, #555396) was used with Alexafluor-labeled goat anti-mouse 568 antibody (from Invitrogen, #A11004).
